# How We Treat Wounds To-Day

**Published:** 1886-05

**Authors:** 


**How We Treat Wounds To-Day: A Treatise on the Subject of Antiseptic Surgery which can be Understood by Beginners.** By ROBERT T. MORRIS, M. D., late House Surgeon to Bellevue Hospital, N. Y.; Member Linnean Society of Natural History, N. Y.; Member of N. Y. County Medical Society. New York and London: G. P. Putnam's Sons. 1886.

To quote the opening words, "This book is modest only in size. It possesses dignity only in its facts. My idea is to present in digestible form a dish of truth from which all bones have been removed. I shall attempt to describe concretely the way in which a few wounds should be treated. Extensiveness will be sacrificed to simplicity." Consequently we have a little book of about 160 pages, from which the young surgeon will learn more about the practical details of Listerism than he can from the great volume. It is in one word a very excellent little book, and worth buying as well as reading.

